# Tissue factor and regenerative medicine: shaping cell and extracellular vesicle-based therapeutic approaches

**DOI:** 10.3389/fcell.2026.1911520

**Published:** 2026-08-03

**Authors:** Van T. Hoang, Quyen Thi Nguyen, Duc Son Le, Lan Anh Thi Ngo, Liem Thanh Nguyen

**Affiliations:** 1 Vinmec Research Institute of Stem Cell and Gene Technology, VinUniversity, Hanoi, Vietnam; 2 Innovation Health Sciences, VinUniversity, Hanoi, Vietnam; 3 Vinmec International Hospital, Hanoi, Vietnam

**Keywords:** cell-based therapy, extracellular vesicle-based therapy, instant blood-mediated inflammatory reactions, regenerative medicine, tissue factor, venous thromboembolism

## Abstract

Regenerative medicine, characterized by breakthroughs in stem cell technology, cell-free therapies, and tissue engineering, holds immense promise for treating various diseases. However, along with the encouraging advancements in translational and clinical research, emerging evidence has demonstrated a potential risk of developing instant blood-mediated inflammatory reactions (IBMIRs) and venous thromboembolism (VTE) with the intravascular delivery of cellular products that do not naturally come into contact with blood. Tissue factor (TF), an initiator of the extrinsic coagulation cascade, plays a pivotal role in these processes. In this comprehensive review, we summarize the biological properties and multifaceted functions of TF in both health and disease. By understanding its physiological and pathological roles, we discuss the implications of TF for IBMIR and VTE in patients receiving cell therapy and extracellular vesicle (EV) therapy. VTEs, including deep vein thrombosis and pulmonary embolism, might have life-threatening consequences. During IBMIR, the recipient’s innate immune cells and humoral coagulation factors drive a hyperacute thromboinflammatory response that traps the infused products, leading to rapid intravascular clearance and compromised therapeutic outcomes. Additionally, we introduce transplantation protocols that demonstrate prevention and monitoring strategies for TF-induced negative effects. Given the increasing complexity of clinical studies and the diverse applications of cell-based therapies, mitigating TF-induced thrombosis is crucial for safer and more effective treatments.

## 1 Introduction

Cell-based therapies represent a transformative approach in modern medicine, broadly classified into stem cell therapies (such as hematopoietic stem cells, mesenchymal stem/stromal cells (MSCs), embryonic stem cells (ESCs), induced pluripotent stem cells (iPSCs), etc.), and other cell therapies (including therapies using immune cells, mononuclear cells, and other differentiated cells, such as hepatocytes, pancreatic cells, and chondrocytes, etc.). Each category plays a distinct role in therapeutic strategies. The clinical translation of MSCs has grown exponentially, with a total number of 55,733 participants by 2022 worldwide (Jovic et al., 2022; Hoang et al., 2025). During the COVID-19 pandemic, the urgency to address severe medical needs spurred approximately 195 trials on cell therapy in just 2 years. The most common cell types include MSCs (72%), natural killer (NK) cells (9%) and mononuclear cells (6%) (Couto et al., 2023).

Additionally, extracellular vesicles (EVs), which are secreted by all cells and act as potent mediators of intercellular communication, are increasingly used. Owing to their ability to transfer proteins, lipids and RNAs, EVs show great promise in modulating disease processes and facilitating tissue repair, making them a novel category in therapeutic applications distinct from those of cellular therapies (Cheng and Kalluri, 2023). Clinical applications of EVs have exponentially expanded, with 103 clinical trials registered since 2011, of which 69 trials have been conducted in the last 4 years (Hoang et al., 2025). 61% of these trials utilized MSC-derived EVs, with the most common indications for COVID-19 and pulmonary diseases.

Although cell-based therapies and EV therapies offer powerful alternatives for regenerative medicine, they also pose significant challenges, particularly with complications such as immediate blood-mediated inflammatory reactions (IBMIRs) and venous thromboembolism (VTE) during intravascular infusion (Moll et al., 2019). IBMIR is an acute, non-specific immune and thrombotic response that occurs within minutes when exogenously transplanted cells comes into direct contact with recipient blood. Primarily triggered by the intravascular delivery of biological products that are naturally sequestered from the bloodstream, IBMIR is characterized by the immediate, coordinated hyperactivation of the host innate immune system, the complement cascade (resulting in cell lysis), and the extrinsic coagulation cascade (leading to platelet activation and rapid clot formation) (Moll et al., 2019). This cascade not only impedes the proper function of the transplanted cells but also may cause broader vascular issues. Consequently, IBMIR poses a substantial clinical risk in both allogeneic and autologous configurations, frequently serving as the upstream driver for a broader, secondary systemic complication: VTE. Unlike the hyperacute local kinetics of IBMIR, VTE typically manifests on a delayed timeline (days to weeks post-exposure). This thromboembolic pathology presents either as deep vein thrombosis, where blood clots form within the deep peripheral venous system, or as a life-threatening pulmonary embolism, which occurs when these thrombi detach, migrate to the lungs and block the blood flow (Lutsey and Zakai, 2023). IBMIR was initially observed in patients receiving allogeneic liver and pancreatic islet transplants (Lee et al., 2016; Bennet et al., 2000). Subsequently, Kanak et al. showed that transplantation of autologous islets can also induce IBMIR, confirming that this reaction occurs independently of classical allo-recognition pathways (Kanak et al., 2014). Growing clinical evidence has since extended these findings, with numerous studies demonstrating an increasing incidence of both IBMIR and VTE in patients receiving MSCs (Moll et al., 2012).

Tissue factor (TF) has been shown to be associated with IBMIR and VTE (Moll et al., 2019; Johansson et al., 2005). TF is expressed by cells surrounding blood vessels to form a hemostatic barrier in the body. Upon injury, TF on the cell surface is exposed to its substrates in blood, including coagulation factor X (FX) and VII (FVII), triggering the extrinsic coagulation cascade. As pancreatic cells, hepatic cells and MSCs express TF to varying extents, intravascular infusion of these cell types can trigger thrombotic events in the recipient. Conversely, targeted blockade of TF suppresses pancreatic islet-induced thrombosis (Johansson et al., 2005). Similarly, TF inhibitory antibodies counteract the active FXa and fibrin formation induced by MSCs (Hoang et al., 2024). TF functions, however, extend far beyond blood clotting, such as its involvement in tumor development and progression, as recent studies have highlighted (Unruh and Horbinski, 2020). Hence, understanding the multifaceted functions of TF is essential both to prevent its negative impact on cell-based therapies and to develop tumor environment-targeted therapies for cancer treatment.

Although the role of TF in IBMIR and VTE incidence has been described in many reports, well-designed studies addressing prevention, early detection or treatment of thrombotic complications are limited (Couto et al., 2023). Notably, the subject is highly complex, as many factors contribute to a given patient’s response to cell-based treatments, such as the individual’s health history and condition at the time of any intervention. In particular, the risk of developing VTE may significantly increase with major surgeries, severe muscle trauma, heart disease, lung disorders, cancer, obesity, other comorbidities and conditions that involve inflammation or increased estrogen levels (Schünemann et al., 2018). Indeed, we observed varying coagulation properties of patients receiving the same batch of allogeneic umbilical cord (UC)-derived MSCs, among which some exhibited significantly increased D-dimer levels, whereas others did not (Hoang et al., 2024). Hence, this review aims to provide a comprehensive overview of the TF protein and its effects on cell- or EV-based therapies. Furthermore, we discuss strategies to prevent IBMIR and VTE, aiming to develop safer and more potent therapies for procoagulant biological products.

## 2 The TF protein: structure, isoforms, and expression

**FIGURE 1:**
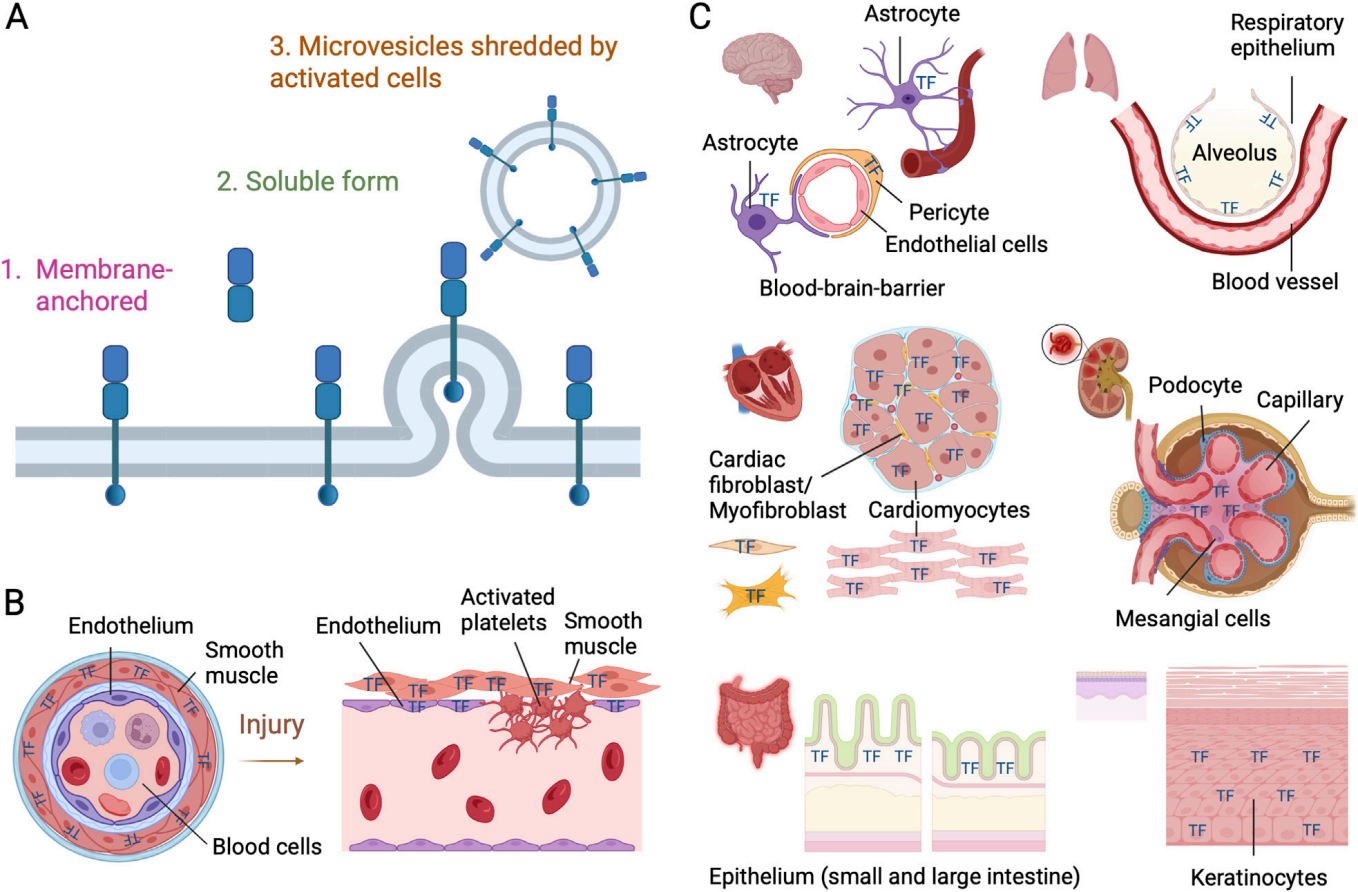
Structural isoforms and physiological distribution of tissue factor (TF). **(A)** Three distinct TF isoforms coexist: (1) plasma membrane-anchored, (2) soluble/alternatively spliced (asTF), and (3) microvesicle-embedded. The full-length plasma membrane-anchored and microvesicle-embedded isoforms consist of an extracellular domain, a single-pass transmembrane domain, and a C-terminal cytoplasmic tail. In contrast, the soluble asTF isoform lacks the transmembrane and cytoplasmic regions, containing only the extracellular domain. **(B)** TF is constitutively expressed at high levels on smooth muscle cells surrounding blood vessels, acting as a perivascular trigger that rapidly initiates coagulation upon damage to the blood vessel wall. **(C)** TF is expressed in many organs, including pericytes and astrocytes in the central nervous system, respiratory epithelial cells in the lungs, myocardiocytes and cardiac fibroblasts in the heart, glomeruli in the renal/splenic architecture, mucosal epithelial cells lining the digestive tract, and keratinocytes in the epidermis. Collectively, this localized distribution forms a defensive “hemostatic envelope” that shields organs from mechanical injury and pathological challenges. Figure created with BioRender.com. Abbreviations: tissue factor (TF).

TF, also known as coagulation factor III, tissue thromboplastin, or CD142, is a 47 kDa transmembrane glycoprotein of the class 2 cytokine receptor family (Figure 1A). (Bazan, 1990) It consists of 263 amino acids organized into three domains: an extracellular domain with residues 1–219, a transmembrane domain ranging from residues 220–242, and a small cytoplasmic domain with 20 amino acids (Spicer et al., 1987). The extracellular domain consists of two immunoglobulin-like domains so that it can act as a receptor for factor VIIa, which functions in the extrinsic coagulation cascade (Harlos et al., 1994). This complex catalyzes the proteolysis of its natural substrates, factors IX and X, upon activation through phospholipids in the membrane (Fiore et al., 1994). The lack of either the extracellular or transmembrane domain, but not the intracellular domain, interrupts its proteolytic activity (Ruf et al., 1991). On the other hand, the intracellular domain is responsible for signal transduction (Ahamed and Ruf, 2004).

TF is expressed in three naturally occurring protein isoforms: (1) plasma membrane-anchored, (2) soluble, and (3) microvesicle-embedded isoforms. Full-length TF is present mainly as a transmembrane protein on the plasma membrane of the cell and is a component of the hemostatic barrier that protects the body and organs from bleeding (Grover and Mackman, 2018). TF is abundantly expressed in vascular muscle cells, perivascular fibroblasts and pericytes surrounding the blood vessel wall (Figure 1B). (Snyder et al., 2013; Cohen et al., 2020) High levels of TF are also observed in astrocytes and pericytes of the brain, epithelial cells of the lung, cardiac fibroblasts and cardiomyocytes of the heart, glomeruli of the kidney, mucosal epithelium, and keratinocytes of the skin (Figure 1C). (Fleck et al., 1990) Under normal conditions, most circulating blood cells and endothelial cells do not significantly express TF. However, monocytes can express TF upon activation during infection and inflammation, whereas the endothelium serves as a critical source of TF in pathological states, such as inflammation or injury (Foley and Conway, 2016; Liu et al., 2004).

Full-length TF can also be integrated into microvesicles under physiological and pathogenic conditions (Figure 1A). (Khaspekova et al., 2016) Platelets, leukocytes, erythrocytes, endothelial cells, and adipocytes release small numbers of TF^+^ microvesicles into the bloodstream (Rank et al., 2018). Moreover, infection-induced sepsis (Aras et al., 2004), myocardial diseases (Suefuji et al., 1997), and cancer (Date et al., 2017) commonly further increase their serum levels. Although the origins of microvesicles depend largely on the disease background, activated monocytes and endothelial cells, as well as tumor cells, are the most common sources (Khaspekova et al., 2016).

The alternative splicing isoform of TF (asTF) can be generated by alternative splicing (Figure 1A). (Bogdanov et al., 2003) Unlike its full-length isotype, asTF is soluble and lacks the transmembrane domain and key residues for interaction with FX. asTF circulates in the blood without coagulation activity until it is exposed to phospholipids, which activate thrombin formation (Censarek et al., 2007). Upon stimulation, asTF is secreted, constituting approximately 30% of the total TF pool in normal human plasma (Bogdanov et al., 2003). The level of asTF is elevated in various diseases, such as sepsis, cancer, diabetes, and cardiovascular and hepatic diseases (Grover and Mackman, 2018).

## 3 Functions of TF

### 3.1 Coagulation

**FIGURE 2:**
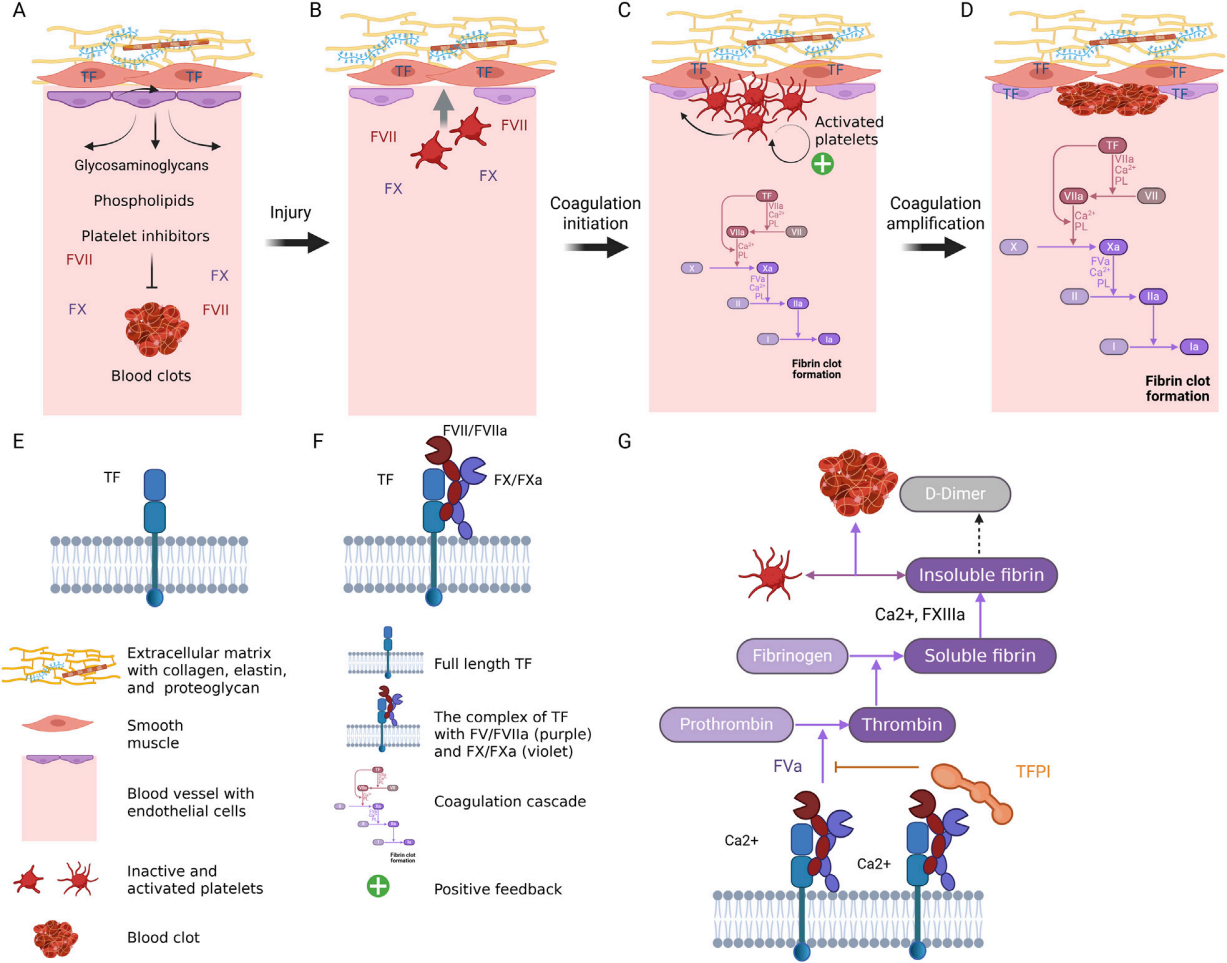
Mechanisms of TF-driven coagulation initiation and clot propagation. **(A)** Under physiological conditions healthy endothelial cells remain non-thrombogenic; they express no surface TF and maintain high levels of endogenous anticoagulant factors, including negatively charged glycosaminoglycans, neutral phospholipids, platelet inhibitors, coagulation inhibitors, and fibrinolysis activators. **(B)** Upon endothelial disruption, subendothelial procoagulant elements, including TF, collagen, and von Willebrand factor (vWF), are exposed to circulating blood components, triggering immediate platelet adhesion and activation. **(C)** Activated platelets undergo rapid degranulation, releasing critical coagulation factors, platelet agonists, adhesion molecules, and local signaling mediators into the microenvironment to propagate the coagulation cascade. **(D)** Cascade factors undergo sequential, reciprocal activation, amplifying the prothrombotic signal to form a localized physical barrier at the injury site. **(E–G)**. Molecular kinetics of the TF complex. **(E)** In the absence of vascular injury or circulating coagulation factors, cell-surface TF remains in a functionally dormant or encrypted state. **(F)** Upon blood vessel injury, exposed TF directly interacts with its clotting factor substrates: FVII, FIX, and FX. **(G)** The TF/FVIIa complex cleaves FX into FXa, which subsequently converts prothrombin into thrombin. Thrombin then cleaves soluble fibrinogen to form fibrin monomers, which self-assemble into protofibril aggregates. This network is covalently cross-linked into stable fibrin clots by FXIIIa in the presence of $Ca^{2+}$ . Figure created with BioRender.com. Abbreviations: calcium (Ca), activated factor V (FVa), activated factor VII (FVIIa), activated factor X (FXa), activated factor XII (FXIIa), tissue factor (TF), tissue factor protein I (TFPI), Willebrand factor (vWF).

Coagulation is a physiological process that protects the body from injury and bleeding. Under homeostatic conditions, endothelial cells secrete many anticoagulant factors, including negatively charged glycosaminoglycans, neutral phospholipids, platelet inhibitors, coagulation inhibitors, and fibrinolysis activators (Figure 2A). Platelets are in a inactive state, whereas circulating coagulation factors, such as platelet-activating factor, clotting factor, prothrombin, fibrinogen, and von Willebrand factor (vWF), remain inactive (Palta et al., 2014). When blood vessels are damaged, platelets are exposed to many coagulation factors, such as TF, collagen, vWF, laminin, thrombospondin, fibrinogen, and vitronectin, in subendothelial tissue (Figure 2B). Platelets become activated and trigger the coagulation cascade (Figure 2C). (Repetto and De Re, 2017) Both platelet agonists released by platelets and thrombin generated during the coagulation process amplify the coagulation cascade and stimulate platelets to form larger aggregates (Figure 2D) (Heemskerk et al., 2002).

TF is crucial for both the initiation and amplification phases of coagulation (Figure 2E), primarily by forming a complex with coagulation factors, including FVIIa, FIXa and FXa (Figure 2F). (Repetto and De Re, 2017) TF is exposed to coagulation factors in the blood during injury, recruiting factor (F)VII/FVIIa to the plasma membrane and enabling interactions between FVIIa and its substrates FIX and FX (Grover and Mackman, 2018). An extrinsic coagulation cascade is thereby initiated. Circulating FVII/FVIIa binds to TF on the cell surface, forming a high-affinity TF–FVIIa complex that recruits and activates FX (Osterud and Rapaport, 1977). Additionally, FVIIa can also activate FIX, and the FIXa/FVIIIa complex further promotes robust FXa accumulation to amplify the cascade (Lu et al., 2004). The protease FXa mediates the proteolysis of prothrombin to generate thrombin. Thrombin cleaves fibrinogen into fibrin and further activates FV, FVIII and platelets during the amplification phase. Fibrin forms stable clots to close the injury site (Figure 2G).

### 3.2 Signal transduction to mediate cell survival, proliferation, migration, and angiogenesis

**FIGURE 3:**
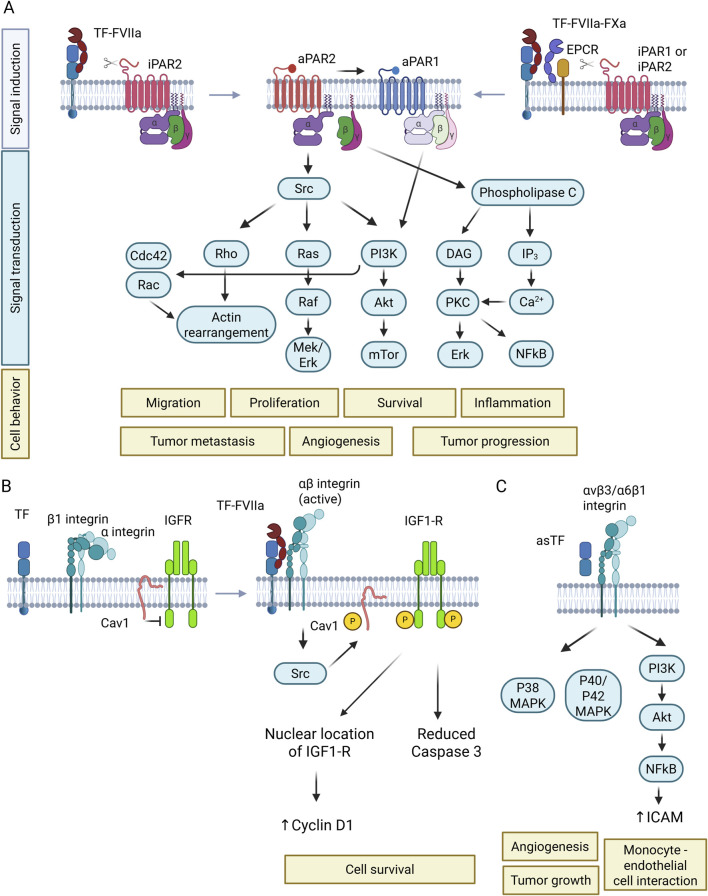
TF-mediated intracellular signaling. **(A)** The TF/FVIIa protease cleaves the inhibitory N-terminal domain of PAR2, a G protein-coupled receptor, enabling receptor self-activation. PAR2 can activate PAR1. The PAR protein family induces many signaling pathways, including the Src, Rho, MAP kinase, PI3K/Akt, Ca^2+^, and NFkB families, which support cell migration, proliferation, and survival as well as regulate inflammation. These processes are involved in angiogenesis, tumor progression and metastasis. **(B)** Full-length TF can send signals into the cell via integrin signaling. In the inactive state, Cav1 inhibits IGF1-R. The TF/FVIIa complex interacts with β1 integrin and changes the conformation of αβ integrin to the active state. αβ Integrin activates Src, which phosphorylates Cav1. Phosphorylated Cav1 is released from IGF1-R so that the tyrosine receptor becomes active, and it translocates into the nucleus to induce cyclin D1 expression. Additionally, IGF1-R activation leads to reduced caspase 3 levels, resulting in increased cell survival. **(C)** asTF activates integrin through a different mechanism from that of the full-length isotype. asTF binds to αvβ3 integrins and α6β1 integrins independently of FVIIa. αvβ3 and α6β1 integrins activate MAPKs, such as p38, p40/42 and PI3K/Akt/NFkB signaling. This signaling pathway impacts angiogenesis, the interaction between monocytes and endothelial cells, and tumor growth. Figure created with BioRender.com. Abbreviations: alternative splicing TF isoform (TF), endothelial protein C receptor (EPCR), factor VIIa (FVIIa), factor Xa (FXa), protease-activated receptor (PAR), and tissue factor (TF).

While the extracellular domain of TF is sufficient to induce hemostasis and thrombosis, the intracellular domain contributes to transducing signals into cells. Mice expressing cytoplasmic domain-deficient TF (TF_ΔCT/ΔCT_) displayed normal coagulant functions (Carson and Bromberg, 2000). However, they are impaired in the recruitment and activation of leukocytes associated with lower levels of proinflammatory cytokines, including TNF-α, IL-1β, and IL-6, and decreased activity of NFkB and mitogen-activated protein kinase p38 signaling (Apostolopoulos et al., 2008). Compared with other members of the cytokine class II receptor family, TF has unique mechanisms for signal transduction, which depend on its proteolytic activity in the TF/FVIIa complex and signaling in the PAR family (Figure 3A). PAR1-activating peptides and PAR2-inhibiting peptides rescued the TF_ΔCT/ΔCT_ phenotype in a mouse model of cardiac infarction (Chong et al., 2021). PARs belong to a seven-transmembrane G protein-coupled receptor family that is activated by proteases such as FVIIa, FXa, and thrombin (Rao and Pendurthi, 2005). When proteases cleave PARs at their N-terminus, the newly exposed N-terminal extracellular domains can serve as ligands for the receptors themselves. The TF/FVIIa complex uses PAR2 as its substrate, whereas the TF/FVIIa/FXa complex activates both PAR1 and PAR2 (Fan et al., 2005). The latter process is mediated by the endothelial protein C receptor (EPCR). EPCR enhances the interaction between FXa and its substrates, PAR1/PAR2, resulting in their proteolysis and activation (Disse et al., 2011). Upon activation by a TF, PAR1 and PAR2 can activate each other and send signals inside the cell (Lin et al., 2013).

Activated PAR subunits can activate many signaling pathways (Figure 3A). (Heuberger and Schuepbach, 2019) It phosphorylates Src family kinases, including c-Src, Lyn, and Yes (Versteeg et al., 2000). Src proteins play multiple functions in the cell, as they activate the MAP kinase cascade (e.g., Erk and p38) to promote cell proliferation, induce PI3K/Akt signaling to favor cell survival, and mobilize the Rho family of GTPases (e.g., Rho, Rac, and cdc42) to reorganize the cytoskeleton (Wojtukiewicz et al., 2015). Activated Rac1 and p38 rearrange actin filaments, hence promoting cell migration (Ott et al., 2005). Moreover, TF-mediated PAR2 activation recruits β-arrestin and ERK1/2 to pseudopodia, promoting local actin assembly and podia extension to mediate cell migration (Ge et al., 2003). PAR2 also stimulates phospholipase C, which induces the generation of the secondary messenger proteins phosphatidyl inositol triphosphate (PI_3_) and diacylglycerol (DAG), resulting in elevated cytosolic Ca^2+^ levels (Versteeg et al., 2000). DAG and Ca^2+^ can activate protein kinase C, which induces MEK-ERK and NFkB signaling (Mackay and Twelves, 2007). In this context, TF can coordinate tumor progression by mediating both cancer cell growth and invasive behaviors (Wojtukiewicz et al., 2015).

Furthermore, TF isoform can transduce signals via the integrin protein family (Figure 3B). (Versteeg et al., 2008a) Full-length TF interacts with β1 integrin via FVII (Rothmeier et al., 2018). Aberg et al. reported that the activated TF/FVIIa/integrin complex activated Src, which might phosphorylate caveolin-1 (Cav1) on tyrosine 14, allowing this protein to release its inhibitory effect on insulin-like growth factor 1 receptor (IGF1-R). IGF1-R was activated and translocated into the nucleus, where it upregulated cyclin D1 expression, favoring cell survival. It also reduces caspase 3 levels, leading to increased resistance to TRAIL-induced apoptosis and promoting survival (Åberg et al., 2020). asTF also interacts with integrins but transduce signals via different mechanisms, as they directly bind to integrins αvβ3 and α6β1 (Figure 3C). Emerging evidence has suggested that asTF-αvβ3 integrin induces endothelial cell migration via PI3K/Akt and p38 MAPK signaling, whereas asTF-α6β1 integrin is involved in capillary formation via the PI3K and p40/p42 MAPK pathways (van den Berg et al., 2009). In cancer, the crosstalk between asTF and β1 integrin actively regulates tumor growth (Kocatürk et al., 2013). Additionally, the TF/integrin complex upregulates the expression of adhesion molecule CAMs on endothelial cells that strengthen monocyte adhesion, allowing their penetration through the blood vessel wall (Srinivasan et al., 2011).

Another role of TF is to promote angiogenesis during embryogenesis and in cancer. TF contributes to the development of embryonic blood vessels, and its inactivation leads to the death of 8.5 days embryos (Carmeliet et al., 1996). Expressing only as little as 1% of the full-length TF-expressing transgene is sufficient to reestablish vasculature integrity and rescue the embryonic death of TF knockout mice (Pawlinski et al., 2002).

High TF levels are correlated with elevated VEGF levels and increased microvessel density in many cancer types (Versteeg et al., 2008a; Khorana et al., 2007). TF overexpression activates TF/FVIIa/PAR2 signaling, inducing the expression of numerous proangiogenic factors, such as VEGF, Cyr61, VEGF-C, CTGF, IL8, MMP-7 and CXCL-1, in cancer cells (Versteeg et al., 2008b). Moreover, the TF/FVIIa complex regulates angiogenesis through endothelial cell migration. The TF/FVIIa/PAR2 complex upregulates the GTPase RhoA and its substrate cortactin in endothelial cells. RhoA phosphorylates and activates cortactin so that this protein can move to the cell periphery and interact with β-actin. This interaction promotes rearrangement of the actin cytoskeleton network, allowing the cell to form lamellipodia and migrate (Zhu et al., 2011). Moreover, asTF ligates with αvβ3 and α6β1 to orchestrate angiogenesis, the first factor is responsible for endothelial cell migration, and the latter promotes the formation of blood vessels (Von Offenberg Sweeney et al., 2005).

Overall, the data suggest that TF plays a significant role in both physiological and pathological contexts. Its classical function in coagulation and thrombosis is well characterized, in which TF loss of function might result in lethality or abnormal clotting. Moreover, the induction of cell signaling through PAR and the integrin protein family allows its impact to go far beyond its role as a coagulation factor, as indicated by TF’s roles in essential cellular processes, including angiogenesis, embryogenesis, and the control of cell proliferation, survival, and migration.

## 4 The impact of TF on cell therapy

**FIGURE 4:**
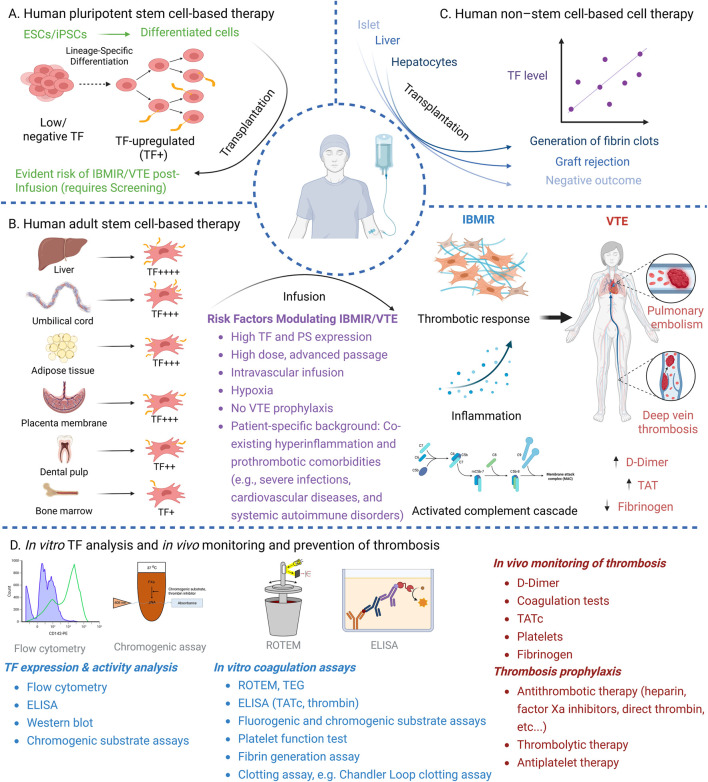
Translational impacts and safety management of TF in cell- and EV-based therapies. **(A)** Pluripotent stem cells, including embryonic stem cells (ESCs) and induced pluripotent stem cells (iPSCs), typically feature baseline low or negative TF profiles. However, they significantly upregulate functional TF expression during lineage-specific differentiation or under pro-inflammatory stimulation. **(B)** MSCs derived from diverse tissue sources exhibit varied, often high levels of surface TF expression, presenting a clinical risk for instant blood-mediated inflammatory reactions (IBMIR) and venous thromboembolism (VTE) upon intravascular administration. **(C)** The transplantation of differentiated cell and solid organs - such as pancreatic islets, liver grafts, or primary hepatocytes - triggers acute intravascular procoagulant events, predisposing recipients to local thromboembolism and subsequent graft rejection. Notably, the clinical severity and magnitude of these adverse reactions correlate positively with the levels of TF on the transplanted grafts. **(D)** To ensure clinical safety, the procoagulant profile of living drugs and cell-free products must be pre-emptively characterized using surface TF and phosphatidylserine (PS) quantification, functional TF activity assays, and *in vitro* coagulation tests (such as ROTEM, TEG, or Chandler loop assays). Thromboprophylaxis regimens (e.g., the administration of direct thrombin inhibitors, factor Xa inhibitors, heparin fractions, antiplatelet therapies, or targeted thrombolytics) should be integrated into clinical protocols based on a systematic, disease background-dependent, and patient-specific risk assessment in case intravascular administrations are applied. Importantly, patients should be actively monitored for early thrombotic safety signals through post-infusion surveillance tracking D-dimer and thrombin-antithrombin complex (TATc) kinetics, global coagulation assays, platelet consumption, and fibrinogen depletion. Figure created with BioRender.com. Abbreviations: embryonic stem cells (ESCs), induced pluripotent stem cells (iPSCs), instant blood-mediated inflammatory reactions (IBMIRs), rotational thromboelastometry (ROTEM), thrombin–antithrombin complex (TATc), thromboelastography (TEG), tissue factor (TF).

Despite year-to-year growth in the clinical application of cell-based therapy in regenerative medicine, many openly question its safety, efficacy, and underlying mechanisms, which are still under investigation. The types of cells utilized in cell-based therapies can be categorized into three groups: pluripotent stem cells, adult stem cells and other cells (El-Kadiry et al., 2021). The following sections delineate the pathophysiological impact of TF across distinct cell-based therapeutic platforms (Figure 4).

### 4.1 Human pluripotent stem cell therapy

Pluripotent stem cells, including ESCs and iPSCs, have emerged as promising therapeutic options in regenerative medicine. However, only a few clinical trials have been conducted on pluripotent stem cells to date, mainly due to ethical concerns, high risks of tumor formation, and technical issues (Hoang et al., 2025). These studies applied retinal pigment epithelium, cardiomyocyte progenitor cells, and oligodendrocyte progenitor cells differentiated from ESCs and iPSCs to treat degenerative diseases of the eye and heart, as well as spinal cord injury (Hoang et al., 2025). Among the limited number of transplanted patients, complications, including IBMIR and thrombosis, have not been reported (McKenna et al., 2022; Menasché et al., 2018; Liu et al., 2018). Moreover, data on TF expression in cell products remain elusive. A few studies have shown that TF is upregulated during ESC differentiation (Figure 4A). During embryogenesis, embryos on day 10.5 require TF expression for the development of the embryonic and extraembryonic vascular systems (Bugge et al., 1996). Furthermore, TF expression is upregulated in trophoblasts and granulocyte–monocyte cells differentiated from human ESCs in correlation with miR-20b downregulation and ERK 1/2 signaling upregulation (Yu et al., 2013). TF enables the enrichment of pancreatic endoderm derived from human ESCs, which give rise to all cell lineages of the pancreas, including functional insulin-producing cells, *in vivo* (Kelly et al., 2011). Endothelial cell-derived iPSCs express low levels of TF, which is strongly enhanced upon TNF-α stimulation (Pflaum et al., 2021). Lysates of megakaryocytes differentiated from iPSCs contain high TF concentrations (Baigger et al., 2018). Accordingly, iPSC-derived platelets express more TF than adult-derived platelets (Krisch et al., 2021). Hence, although ESCs and iPSCs might exhibit a limited level of TF, their differentiated progenies might upregulate TF expression, which should be considered in safety analyses for clinical applications.

### 4.2 Human adult stem cell therapy

Although only MSCs have been reported to be associated with thrombogenic events, other stem cells used in therapies could also pose thrombotic risks depending on the manipulation before infusion, the cell state, and the patient’s condition (Figure 4B). Hematopoietic stem cells and their progenies are components of the blood system and express no TF under physiological conditions. However, they can express TF when activated, particularly in pathological conditions or certain inflammatory states (Foley and Conway, 2016). Data on TF-induced pathogenic coagulation in other multipotent stem cell systems, such as neural stem cells, limbal stem cells, cardiac stem cells, and skin stem cells, are still lacking. This could be related to the low patient numbers in current clinical studies or their local applications to reduce the risk of blood exposure.

Since the first emergence of MSCs in the 1950s, thousands of clinical trials have been conducted across the globe to date because of their unique characteristics: (1) ease of acquisition with no ethical barrier; (2) immunomodulatory properties with low MHC class I expression and negative MHC class II expression; and (3) regenerative functions via a wide range of growth factors, cytokines, chemokines and EVs (Hoang et al., 2022). While many clinical trials have supported the safety profiles of MSCs in treating various human diseases, it is essential to acknowledge existing safety concerns. Notably, thrombotic events have been observed in response to MSC administration. As MSCs are frequently found in tissues surrounding blood vessels, they express variable levels of TF/CD142 and can also secrete it into their microenvironment. Hence, intravascular infusion of MSCs without proper anticoagulant prophylaxis can cause mild-to-severe thromboembolic adverse events in patients (Caplan et al., 2019). For the first time, Bennet and Moberg described the IBMIR, which is associated with activation of the coagulation and complement systems, resulting in graft failure and TF/CD142-induced thrombosis upon pancreatic islet transplantation (Bennet et al., 2000; Moberg et al., 2002). Approximately 10 years after these reports were published, the death of a Korean patient who received autologous adipose-derived MSCs following pulmonary embolism in 2010 attracted attention to uncontrolled stem cell treatments (Cyranoski, 2010). In 2012, Moll et al. reported that *in vitro* culture of MSCs markedly increased the expression of TF on the cell surface of MSCs, which caused IBMIR *in vivo* (Moll et al., 2012). Intravenous infusion of cultured adipose stem cells induced pulmonary embolism and mortality in 85% of the treated mice within 24 h after administration. The coagulant activity of these cells was suppressed by preconditioning with an inhibitory TF antibody or via the use of FVII-free plasma, suggesting a connection between the TF/FVIIa complex and MSC-induced thrombogenic events (Moll et al., 2012; Hoang et al., 2024; Tatsumi et al., 2013).

While quantifying tissue factor (TF) expression provides valuable phenotypic data, surface antigen levels do not necessarily correlate with the functional procoagulant activity of cell products (Hoang et al., 2024). This discrepancy is governed by two primary mechanisms. First, the thrombogenicity of TF depends heavily on its conformational state, shifting from an inactive (encrypted) form to a highly active (decrypted) form upon stimulation (Chen and Hogg, 2013; Langer and Ruf, 2014). Second, the absolute procoagulant profile is a multi-parametric phenomenon. Cell products often expose varying levels of other critical procoagulant factors on their surface, most notably phosphatidylserine (PS), which can act synergistically with TF to accelerate the extrinsic coagulation cascade. Consequently, several functional *in vitro* coagulation assays have been developed to predict thrombogenic risk of cellular therapeutics (Table 1). Some studies have used viscoelastometric methods, including thromboelastography (TEG) and rotational thromboelastometry (ROTEM), to measure viscoelastic changes due to fibrin polymerization in the presence of MSCs (Oeller et al., 2018; Wu et al., 2022; Araldi et al., 2022). Fluorogenic and chromogenic substrate assays are also widely used to quantify TF, thrombin-antithrombin complex (TATc) or thrombin levels (Hoang et al., 2024; Wu et al., 2022). Other methods, such as the platelet function test, whole-blood Chandler loop clotting assay, fibrin generation assay, and clotting assay, can also be applied (Moll et al., 2012; Netsch et al., 2018; Gleeson et al., 2015). Collectively, these assays enable a comprehensive analysis of the coagulant activity of therapeutic products, mapping out specific clinical and manufacturing risk factors that govern MSC-induced thrombosis in patients (Table 2).

**TABLE 1:** Methods used to evaluate the coagulant activity of mesenchymal stem/stromal cells (MSCs).

No.	Publication	Target population	Method
1	Moll et al. (2012)	Human bone marrow (BM)-MSCs	- Whole-blood Chandler loop clotting assay - ELISA (quantify thrombin-antithrombin complex (TATc), activated FVII antithrombin complex (FVIIa-AT), FXI-AT, FXII-AT, and complement activation makers C3a and sC5b-9)
2	Tatsumi et al. (2013)	Mouse adipose tissue-derived MSCs *(*AT-MSCs)	- Rotational thromboelastometry (ROTEM) - Plasma clotting assay
3	Gleeson et al. (2015)	Female Landrace pigs BM-MSCs	- ELISA (quantify TF)
4	Christy et al. (2017)	Human AT-MSCs, BM-MSCs	- Thromboelastography (TEG) - Calibrated automated thrombogram (CAT)
5	Netsch et al. (2018)	Human BM-MSCs, lipoaspirate MSCs, cord blood MSCs, and human umbilical vein endothelial cells (HUVECs)	- Platelet function test - Light transmission aggregometry (LTA)
6	Oeller et al. (2018)	Human AT-MSCs, BM-MSCs, and umbilical cord-derived MSCs (UC-MSCs)	- ROTEM - Plasma clotting assay
7	Perlee et al. (2019)	Mice infection with *K. pneumoniae* and mice infused with human AT-MSCs	- ELISA (quantify thrombin-antithrombin complexes - TATc)
8	Araldi et al. (2022)	Human immature dental pulp stromal cells	- ROTEM
9	Wu et al. (2022)	Rat AT-MSCs, BM-MSCs	- ROTEM
10	Hoang et al. (2024)	Human AT-MSCs, BM-MSCs, DP-MSCs, and UC-MSCs	- Plasma clotting assay - ELISA (FXa quantification assay)

Abbreviations: adipose tissue-derived mesenchymal stem/stromal cells (AT-MSCs), anti-thrombin (AT), bone marrow-derived mesenchymal stem/stromal cells (BM-MSCs), calcium (Ca), calibrated automated thrombogram (CAT), dental pulp (DP), factor VIIa (FVIIa), factor Xa (FXa), factor XI (FXI), factor XII (FXII), human umbilical vein endothelial cells (HUVECs), light transmission aggregometry (LTA), mesenchymal stem/stromal cells (MSCs), rotational thromboelastometry (ROTEM), thrombin–antithrombin complex (TATc), thromboelastography (TEG), tissue factor (TF), umbilical cord-derived mesenchymal stem/stromal cells (UC-MSCs).

**TABLE 2:** Comparative overview of coagulation assays for cellular/biological products.

Method	Principle	Strengths	Limitations	Relevance to product testing
CAT	Fluorogenic substrate cleaved by thrombin in plasma; fluorescence measured continuously over time	Full kinetic profile of thrombin generation; sensitive to both pro- and anticoagulant shifts; works in platelet rich plasma (PRP) or platelet poor plasma (PPP)	Requires specialized software (Thrombinoscope); plasma-based only	Detects if cellular products express TF or PS, triggering coagulation; ideal for procoagulant risk assessment
Chandler Loop Clotting Assay	Blood or plasma circulated in a closed tubing loop rotating at controlled speed to mimic blood flow; contact with test material assessed	Mimics physiological flow conditions; can use whole blood	Not highly standardized; variable results between labs; technically demanding setup	Excellent for testing if devices, biomaterials, or cell-based products trigger thrombus formation under flow conditions - closely mimics *in vivo* blood contact
ELISA (Biomarkers: complements C3a, sC5b-9, FVIIa-AT (extrinsic pathway), FXa (common pathway), FXI-AT (intrinsic pathway), FXII-AT (contact activation), TATc (thrombin-antithrombin complex, thrombin generation), TF expression)	Antibody-based sandwich ELISA quantifies specific soluble activation biomarkers in plasma after product exposure	Highly specific and quantitative; pathway-specific biomarkers; suitable for mechanistic investigation; high sensitivity	Requires multiple individual assays for full panel; pre-analytical handling critical; does not capture real-time kinetics	Mechanistic study to analyze which coagulation or complement pathway is activated by the product
Plasma Clotting Assay	Test product incubated with plasma (PPP or PRP); clot initiation measured optically or mechanically via PT, aPTT, turbidimetry, or observation of macroscopic thrombus formation	Simple, widely standardized if PT and aPTT are used; low cost; available in most clinical/research labs	Plasma-based only; does not capture cellular contributions	Detects whether product shortens or prolongs clotting time; screens for procoagulant or anticoagulant activity
Platelet Function Test	Platelet activation or aggregation assessed by flow cytometry (surface markers) or light transmission aggregometry (LTA) after product exposure	Directly assesses platelet response; multi-readout platform available: flow cytometry is highly sensitive, LTA is quantitative and well-validated	Platelets are fragile and time-sensitive; results vary with anticoagulant used; fresh blood required	Determines if product induces platelet activation or aggregation, a key thrombogenicity risk for cell therapies and devices
ROTEM	Whole blood placed in a cuvette; a rotating pin measures clot viscoelastic resistance over time	Uses whole blood; captures full hemostatic process including fibrinolysis	Operator-sensitive; expensive equipment; limited standardization across labs; fresh whole blood required	Assesses overall clot formation and lysis triggered by test product in whole blood; useful for biomaterials and cell therapies
TEG	Similar to ROTEM; whole blood in a cup with an oscillating pin measuring clot viscoelastic properties	Global hemostasis assessment in whole blood; real-time clot kinetics and lysis; widely used clinically	Similar to ROTEM; results not directly interchangeable with ROTEM; fresh whole blood required	Evaluates whether product accelerates or inhibits clot formation

Abbreviations: anti-thrombin (AT), calibrated automated thrombogram (CAT), factor VIIa (FVIIa), factor Xa (FXa), factor XI (FXI), factor XII (FXII), light transmission aggregometry (LTA), platelet rich plasma (PRP), platelet poor plasma (PPP), phosphatidyl serine (PS), rotational thromboelastometry (ROTEM), thrombin–antithrombin complex (TATc), thromboelastography (TEG), tissue factor (TF).

The biological nature and inherent heterogeneity of MSCs play a decisive role in their therapeutic efficacy and safety profile post-infusion (Shokoohmand et al., 2026). Because MSCs constitute highly heterogeneous cell populations, and their therapeutic properties and biological features vary significantly based on donor characteristics, tissue origin, isolation method, and *in vitro* culture conditions. MSCs can be isolated and expanded from many tissue sources, including bone marrow (BM), adipose tissue (AT), UC, placental membrane (PM), and dental pulp (DP). Notable differences in baseline TF expression exist among these distinct sources. For instance, strong TF expression has been observed in both AT-MSCs and UC-MSCs, followed by intermediate levels in DP-MSCs, whereas the lowest baseline TF expression is consistently detected in BM-MSCs (Hoang et al., 2024; Oeller et al., 2018; Moll et al., 2015). Importantly, the procoagulant risk of MSCs is not merely an intrinsic biological trait of the source tissue; it is fundamentally shaped by upstream manufacturing parameters and processing conditions. Higher passage numbers, prolonged expansion, and exposure to specific media supplements like animal-derived serum or human platelet lysates, might progressively upregulate surface TF expression (Moll et al., 2022).

These disparate expression profiles correlate directly with *in vivo* safety outcomes. Shiratsuki et al. demonstrated that the survival rate at 24 h in mice administered AT-MSCs was only 46.4%, whereas it was 95.5% in the BM-MSC-infused group. Consistently, the prothrombin time was markedly longer in the former group, suggesting that AT-MSC-induced thrombi led to decreased serum levels of coagulation factors and increased mouse mortality (Shiratsuki et al., 2015). Another study from Moll et al. reported that placental decidua-derived MSCs (PD-MSCs) exhibited stronger hemostatic properties and coagulant activity than BM-MSCs did (Moll et al., 2015). Therefore, the choice of MSC type and product specifications are critical, especially for systemic administration in coagulopathic patients (Christy et al., 2017). Araldi et al. selected DP-MSCs with less than 25% TF^+^ cells for treating COVID-19 patients (Araldi et al., 2022). Despite the elevated risk of VTE in severe COVID-19 cases, this stringent cell-selection strategy, combined with concomitant heparin prophylaxis, proved to be clinically safe.

Notably, TF-deficient HUVECs still exhibited *in vitro* procoagulant activity due to PS surface exposure (Araldi et al., 2022). In our study, approximately 5%–15% of DP- and UC-MSCs were positive for PS. Coculture of MSCs with an inhibitory antibody effectively blocked TF-induced FXa activation but only partially reduced MSC-induced coagulation (Hoang et al., 2024). These findings highlight that while TF is an important player, other factors also contribute to coagulation and thrombosis upon MSC administration. Particularly, cryopreservation and thawing process can lead to dramatic exposure of PS by apoptotic cells, cellular fragments, or shed apoptotic bodies to coagulant factors in blood, leading to thrombogenesis (Shokoohmand et al., 2026).

In contrast to many studies reporting MSC-induced coagulation, Netsch et al. demonstrated the anticoagulant effects of MSCs from BM, AT, and umbilical cord blood, in which they inhibited the agonist-induced activation and aggregation of platelets in platelet-rich plasma and patient whole blood (Netsch et al., 2018). The underlying mechanism is the high expression of CD73 on MSCs, which converts platelet-released ATP and ADP into adenosine, leading to its accumulation at an inhibitory level. Adenosine increases the synthesis of cyclic adenosine monophosphate (cAMP) and the phosphorylation of vasodilator-stimulated phosphoproteins in platelets, which stops the activation cascade. The antithrombogenic property of BM-MSCs was further demonstrated when MSCs were seeded into a biodegradable nanofibrous scaffold with aligned nanofibers. When the scaffold alone induced platelet activation/adhesion, the presence of MSCs diminished this process (Hashi et al., 2007). Thus, the pro- and anticoagulant effects of MSCs might be due to differences in experimental models, cell sources, and functional readouts, and further studies are needed.

This multi-parametric risk profiling is increasingly critical when interpreted within the broader context of the post-administration fate of exogenously delivered MSCs. Following systemic intravenous administration, a substantial fraction of MSCs encounter an immediate blood-contact reaction involving simultaneous coagulation, complement, and innate immune activation (Shokoohmand et al., 2026). This interaction not only influences thromboembolic risk but plays a decisive role in the active clearance, entrapment, and ultimate survival of infused cells. Hoang et al. reported that patients received intravenous UC-MSC infusion exhibited increased D-dimer levels at 24 h, whereas those who received cells via the intrathecal route did not (Hoang et al., 2024). Furthermore, a high dose of administered MSCs is strongly associated with a greater risk of coagulation, which can be reversed by TF-blocking antibodies (Christy et al., 2017; George et al., 2018). Hence, administration route, cell dose, infusion rate, and formulation buffer compositions can play an important role in induction of the coagulation cascade.

It is also crucial to emphasize that while *in vitro* assays provide valuable insight into the basal hemocompatibility of MSCs, they remain insufficient for predicting complex procoagulant behavior post-administration. MSCs function as dynamic, living therapeutics, their vascular safety profile is heavily modulated by the host microenvironment (Foley and Conway, 2016). Consequently, evaluating product safety requires a holistic approach that integrates both the intrinsic properties of the cellular product and the recipient’s underlying pathophysiological condition, while fully acknowledging baseline patient heterogeneity. This clinical heterogeneity represents one of the most challenging variables in regenerative medicine, directly driving both the risk of adverse hemocompatibility events and the variability in therapeutic efficacy observed across clinical applications.

Taken together, understanding TF-mediated hemocompatibility is therefore essential not only for optimizing safety profiles but also for deciphering how post-administration biology determines the localized clearance versus the functional persistence and therapeutic mechanism of the living drug. Other factors, such as PS, present on the MSC surface might also be responsible for thrombotic events (Hoang et al., 2024). Thus, one needs to consider the detrimental effects of TF and other potential procoagulant factors as well as develop appropriate strategies to control the incidence of IBMIR and VTE for safer therapies.

### 4.3 Non-stem cell-based cellular therapies and solid organ transplantation

The expression of TF presents significant translational challenges for non-stem cell-based cellular therapies and solid organ transplantation, primarily by driving thromboinflammatory complications that compromise graft survival.

The transplantation of islets of Langerhans represents a vital therapeutic strategy for type 1 diabetes; however, TF expression on pancreatic islet cells is heavily associated with poor clinical outcomes (Figure 4C). (Bertuzzi et al., 2004) Islet-induced TF rapidly activates the host coagulation cascade, triggering detrimental thrombotic reactions in recipients (Moberg et al., 2002). Other studies have shown that the expression of TF on islet cells is a key factor in this process. It activates the IBMIR, leading to the binding of platelets to the islet surface, infiltration of leukocytes into the transplant, and consequently disruption of islet integrity and islet loss (Naziruddin et al., 2014). Consequently, implementing targeted strategies to modulate TF expression and mitigate IBMIR and subsequent VTE is essential to improving the long-term success of clinical islet transplantation.

Similarly, hepatocyte transplantation offers a promising alternative to whole-organ liver transplantation for acute liver failure and specific inherited metabolic disorders. However, its clinical efficacy is restricted by the fact that approximately 70% of transplanted hepatocytes die immediately following infusion, with IBMIR identified as a primary mediator of this early cell loss (Lee et al., 2016). Infused hepatocytes secrete and express functional TF, initiating local thrombin generation and subsequent fibrin clot formation. This generated thrombin further activates host complement proteins, culminating in the assembly of the membrane attack complex and direct hepatocyte lysis. To counteract this early cell attrition and enhance functional engraftment, current research is focusing on multi-modal strategies, including systemic anticoagulation, microencapsulation techniques, and novel membrane-bound cytoprotective agents designed to locally inhibit complement and coagulation factors.

Beyond primary grafts, TF expression is highly inducible upon inflammatory or mechanical stimulation on endothelial cells (Parry and Mackman, 1995), monocytes/macrophages (Osterud, 1998), and monocyte-derived dendritic cells (Kasuda et al., 2019). For instance, endothelial progenitor cells markedly upregulate TF under inflammatory conditions, conferring an elevated thrombin generation capacity that introduces a distinct prothrombotic risk to endothelial progenitor cell-based therapies (Cuccuini et al., 2010).

In pathological microenvironments, infiltrating lymphocytes have also been suggested to modulate localized TF expression - such as in clear cell carcinoma - thereby influencing VTE rates (Gi et al., 2021). These dynamics indicate that TF can contribute to the immunosuppressive architecture of the tumor microenvironment while actively altering the function and trafficking of immune cells. Accordingly, cell-based therapies must be carefully evaluated to limit the adverse impacts of TF on clinical safety and efficacy.

In the context of solid organ transplantation, elevated TF expression within liver tissue has been strongly correlated with acute and chronic graft rejection compared to non-rejecting controls (Usui et al., 2009). Additionally, enhanced TF expression, together with upregulated plasminogen inhibitor-1 expression via C-reactive protein, activates prothrombotic pathways, leading to early vein graft thrombosis after coronary artery bypass surgery (Ji et al., 2014).

Collectively, the regulated expression of TF across these diverse cellular platforms exerts predominantly prothrombotic effects on non-stem cell-based therapeutics and solid organ transplantation. Further research remains imperative to fully map TF expression kinetics and develop localized interventions capable of preserving treatment efficacy under varied pathological conditions.

## 5 The impact of TF on EV-based therapy – an understudied issue

Procoagulant EVs are secreted by many cells under physiological and pathological circumstances (Table 3). Compared with those carrying phospholipids (e.g., PS), TF-expressing EVs are found in a small proportion of circulating EVs (Ye et al., 2022). However, phospholipids can activate TF through a process known as decryption, which induces conformational changes and enhances its coagulant activity (Chen and Hogg, 2013; Langer and Ruf, 2014). Hence, TF and PS might act synergistically to induce the coagulation cascade.

**TABLE 3:** TF-expressing EVs across physiological, pathological, and regenerative medicine contexts. TF-expressing EVs are constitutively or inducibly shed by a wide array of cell types and have been increasingly identified within cell-free therapeutic products. Notably, both cellular TF expression and the shedding of TF-expressing EVs are significantly upregulated during pathological states, such as systemic infections and malignancies, where they directly correlate with an elevated incidence of venous thromboembolism (VTE) and poor clinical outcomes.

No.	Publication	Target population	Origin of EVs	Findings
Studies of therapeutic EV products				
1	Zhu et al. (2022)	Mouse model for bronchopulmonary dysplasia (BPD)	Human amniotic epithelial cells (hAECs)	- Full-term hAEC-derived EVs displayed a higher surface expression of TF and CD133, induced more pronounced macrophage phagocytosis, but lower T-cell suppression than preterm hAEC-EVs. - Although both EVs reduced inflammation on postnatal day 7, only the first ones increased numbers of type II alveolar cells and improved lung functions
2	Silachev et al. (2019)	Healthy women 25–30 years old who delivered healthy full-term infants by cesarean section (n = 6)	Human UC-MSC-derived EVs	- UC-MSC secreted EVs exhibited procoagulant effects on platelet-free plasma. - Treatment of children with UFH did not prevent MSC induced clotting activity on their blood. - UC-MSC derived EVs was lack of TF, but PS is expressed on ca. 3% EVs
Studies of physiological EVs				
1	Hu et al. (2022)	- Healthy breastfeeding women (n = 6) - Healthy women undergoing routine amniocentesis (n = 7)	EVs from milk, saliva, urine, and amniotic fluid	- EVs of body fluids including milk, amniotic fluid, saliva and urine initiated clotting in FVII‐deficient plasma that was blocked by an anti‐FVII antibody and partially by an anti‐TF antibody. - TF/FVIIa of EVs from body fluids activated FXa to take part in hemostatic protection
2	Nakatani et al. (2022)	*In vitro* study	3T3-L1 Adipocyte-derived EVs	- Adipocyte-derived EVs mediated clotting in plasma that was enhanced by TNF-α. - Blocking of TF and PS diminished the adipocyte-derived EV-induced clotting
3	Ansari et al. (2021)	C57BL/6J mice	Perivascular cells, e.g., fibroblasts and vascular smooth muscle cells	- HNE increased TF + microvesicles and levels of inflammatory cytokines in blood. - The drug interrupted endothelial cell barrier integrity allowing TF + microvesicles to enter the blood stream. - The use of inhibitory anti-TF antibody blocked HNE induced thrombosis and inflammation
4	Hohensinner et al. (2020)	Healthy donors (n = 75)	Macrophage-derived EVs	- Unlike monocytes, stimulation of macrophages with LPS and IFN-γ did not increase TF expression, as TF promoter was hypermethylated. - Polarization of macrophages with IL-4 and IL-13 was associated with higher TF production and activity and elevated TF^+^ EV release, which was induced by activation of transcription-6 signaling and poly ADP ribose polymerase
5	Rothmeier et al. (2019)	Cell line	Endothelial cells	- FVIIa/integrin β1 activated PAR2 signaling that directed TF to endosomal compartments and inhibited its release on EVs. - TF/FVIIa/FXa activated PAR2 signaling that induced quick increase of highly procoagulant TF + EVs followed by production of TF-inactive EVs with lower coagulant activity
6	Connolly et al. (2018)	Healthy donors (n = 7)	EVs in plasma	- Circulating EVs were mainly derived from platelets, leukocytes, erythrocytes, and endothelial cells. - Adipocyte-derived EVs were also detected that expressed adipocyte proteins and adipokines
7	Rank et al. (2018)	Healthy stem cell donors (n = 12)	BM and peripheral blood	- High numbers of EVs were present in BM and peripheral blood. - BM EVs were derived from erythrocytes (43.2%), megakaryocytes/platelets (27.6%), leukocytes and progenitor cells (25.7%), endothelial cells (2.0%), and hematopoietic stem cells (1.5%). - Peripheral blood EVs were originated from platelets (93.9%), leucocytes (4.5%), erythrocytes (1.8%), endothelial cells (1.0%), and hematopoietic stem cells (0.7%). −1.3% bone marrow EVs and 0.9% peripheral blood EVs were positive for TF.
8	Yu et al. (2018)	Healthy human volunteers (n = 5)	Human saliva-derived EVs	- Human salivary EVs exposed coagulant TF, CD24, a ligand of P-selectin, but not PSGL-1. - TF+/CD24+ salivary EVs bind to activated platelets and trigger coagulation
Studies of pathological EVs during virus infections				
1	Girard et al. (2023)	- Patients infected with SARS-CoV-2 (n = 108) - Healthy donors (n = 24)	Plasma	- EVs were 9-fold elevated in COVID-19 patients than healthy donors. −41% of COVID-19 patients’ plasma samples contained high D-dimer levels; of these, 19% samples demonstrated EV TF specific coagulant activity. - EV increased thrombin generation in anti-TF-treated and FXI-deficient plasma suggesting that this was mediated via a TF-independent mechanism, probably through phospholipid
2	Burrello et al. (2022)	Patients infected with SARS-CoV-2 (n = 261)	Serum	- Pro-coagulant TF^+^ EVs was increased in response to inflammation following SARS-CoV-2 infection. - TF-EVs linked to COVID-19 patient outcome and mortality. - The cytokines storm and the hyperactivation of platelets may contribute to increase the release of decrypted TF-EVs with high pro-coagulant activity
3	Campello et al. (2022)	Patients infected with SARS-CoV-2 (n = 91)	Plasma (mostly from platelets, endothelial cells, leukocytes, and neutrophils)	- COVID-19 patients exhibited significantly higher numbers of EVs. - EVs derived from leukocytes, endothelium, and pericytes of healthy donors expressed TF and its expression was increased in COVID-19 patients. - The presence of PS, Calcein, E-Selectin, P-Selectin, ACE2, and CD45 on EVs correlated with phospholipid-dependent clotting time *in vitro*, but TF and PDGF-β levels did not. - High plasma levels of platelet-derived EVs at baseline may be associated with VTE.
4	Balbi et al. (2021)	- Patients infected with SARS-CoV-2 (n = 34) - Healthy donors (n = 16)	Serum (mostly from endothelial cells and platelets)	- EVs from COVID-19 patients strongly expressed TF, CD49e, CD209, CD86, CD69, CD133/1, and CD20. - TNF-α was directly correlated with the TF expression on EVs, while arterial blood oxygenation negatively impacted its level. - COVID-19 patients with high TF-EV levels had poor outcome
5	Rondina et al. (2016)	Patients with influenza A/H1N1 infection (n = 15)	Plasma	- Level of TF^+^ microvesicle activity and IL-8 were correlated with mortality in H1N1-infected patients, while other cytokines, thrombin-antithrombin complexes, and D-dimer did not
Studies of pathological EVs in cancer				
1	Haag et al. (2022)	- Patients with cholangiocellular carcinoma receiving selective internal radiotherapy (n = 47) - Healthy donors (n = 12)	Plasma	- TF expression was significantly increased on EVs derived from cholangiocellular carcinoma patients compared to healthy donors - EVs might be secreted from different subsets of leukocytes including T cells and antigen presenting cells, endothelial cells, and platelets. - Selective internal radiotherapy tended to reduce TF expression on EVs
2	Tawil et al. (2021)	Glioblastoma	The glioma cell lines U373P and U87P	- Glioma cell lines secreted TF^+^ EVs. - Injection of glioma-derived TF^+^ EVs initiated thrombotic events in correlation with increased D-dimer level in mice
3	Sasano et al. (2021)	Ovarian cancer	The ovarian cancer cell lines CAOV3 and OVCAR8	- TF^+^ ovarian cancer cell-derived EVs mediated platelet aggregation *in vitro* and thrombosis in mice
4	Mitrugno et al. (2019)	- Patients with colon cancer (n = 39,862) and controls (n = 10,321,311) - Human colon adenocarcinoma cell line SW480	Colon cancer cells and platelets	- VTE occurred 4.5 times more frequently in colon cancer patients than healthy people. - Colon cancer cells activated PAR4 signaling in platelets depending on TF and thrombin, resulting in platelet activation, their secretion of EVs, and fibrin formation
5	Van Es et al. (2018)	Patients with advanced cancer (n = 648)	Plasma	−6.1% advanced cancer patients developed VTE. - High EV-TF activity increased risk of developing VTE, particularly in those with pancreatic cancer
6	Stark et al. (2018)	Pancreatic cancer	Plasma	- Pancreatic adenocarcinoma cells released microparticles that induced thrombi in mice. - This activity was caused by exposure of phosphatidylethanolamine on microparticles that activated FXa leading to deep vein thrombosis
7	Bang et al. (2016)	- Patients with ischemic stroke and active cancer (n = 155), with ischemic stroke only (n = 25), with cancer only (n = 32) - Healthy donors (n = 101)	Serum	- The levels of cancer cell-derived CD326^+^TF^+^ EVs were higher in cancer-related stroke than in the other groups. *-* Cancer cell-derived EVs may cause cancer-related coagulopathy independent of the levels of TF^+^ EVs
8	Woei-A-Jin et al. (2016)	Pancreatic cancer	Plasma	- Pancreatic adenocarcinoma patients with VTE showed increased microparticle-associated TF activity in plasma. The highest activity was observed in those with severe thrombosis and cerebral embolism despite anticoagulant therapy. - A high microparticle-associated TF activity was associated with metastasis incidence and worse outcome of patients. - Macrophages in the stroma surrounding adenocarcinoma might be the source of these microparticles
9	Manly et al. (2010)	Cancer patients with and without VTE (n = 53 and 13, respectively)	Plasma	- Cancer patients with VTE showed higher TF activity on microparticles compared to the ones without VTE.
10	Yu et al. (2005)	Colorectal cancer	The human colorectal cancer cell lines HCT116 and DLD-1	- K-ras and p53 controlled TF expression in human colorectal cancer cells, probably via MAPK and PI3K signaling. - TF expression and the level of TF-associated EVs were determined by genetic status of cancer cells

Abbreviations: 4-hydroxy-2-nonenal (HNE), acute respiratory syndrome coronavirus 2 (SARS-CoV-2), adenosine diphosphate (ADP), bone marrow (BM), extracellular vesicles (EVs), factor VIIa (FVIIa), factor Xa (FXa), interferon gamma (IFN-γ), interleukin (IL), lipopolysaccharide (LPS), mitogen-activating phosphate kinase (MAPK), mesenchymal stem/stromal cells (MSCs), protease-activated receptor 2 (PAR2), platelet-derived growth factor (PDGF), phosphatidylserine (PS), tissue factor (TF), umbilical cord (UC), and venous thromboembolism (VTE).

EVs from body fluids, including milk, amniotic fluid, saliva and urine, induce clotting in FVII-deficient plasma, which is blocked by anti‐FVII and anti‐TF antibodies (Hu et al., 2022). These findings suggest that EVs from body fluids secrete TF/FVIIa to activate FX in plasma, forming a hemostatic barrier during bleeding. EVs are present in high numbers in the BM and peripheral blood, of which 1.3% and 0.9% of EVs carry TF, respectively (Rank et al., 2018). Platelets, erythrocytes, leukocytes, and endothelial cells are the main sources of circulating EVs in the blood (Zarà et al., 2019). The release of procoagulant TF on EVs is regulated through PAR2 signaling in endothelial cells. Upon PAR2 activation, endothelial cells quickly release highly thrombogenic EVs, followed by an increase in the number of TF-encrypted EVs in the later phase, which results in lower coagulant activity (Rothmeier et al., 2019). Tripisciano et al. reported that platelet-derived EVs induce fibrin clots depending on the dose and exposure to PS, whereas TF plays only a minor role. Unstimulated monocytic EVs exhibit low coagulant activity, but this activity quickly increases upon stimulation with LPS, which activates TF and consequently the downstream coagulation process (Tripisciano et al., 2017). The proinflammatory cytokine interleukin-33 also induces monocytes to upregulate TF expression and activity and their release of circulating procoagulant microvesicles in patients with cardiovascular diseases (Stojkovic et al., 2017).

In addition to blood cells, other cell types can serve as abundant sources of TF-bearing EVs. A fraction of circulating TF^+^ EVs originate from adipocytes (Connolly et al., 2018). Mature adipocytes produce procoagulant EVs without additional stimuli, but their coagulation activity increases significantly in the presence of TNF-α. This activity can be diminished by blocking TF and PS (Nakatani et al., 2022). The release of TF^+^ microvesicles by fibroblasts and vascular smooth muscle cells can be induced by the oxidative stress product HNE (Ansari et al., 2021). The drug also disrupts the endothelial cell barrier, allowing TF^+^ microvesicles to enter the bloodstream. Blocking TF inhibited both thrombin generation and the inflammatory response in treated mice (Ansari et al., 2021).

While the role of endogenous, circulating TF^+^ and PS^+^ extracellular vesicles as pathogenic biomarkers in cancer, sepsis, and viral infections is well established, evaluating the hemocompatibility of isolated or engineered therapeutic EV products represents a critical, understudied subject in regenerative medicine. Mechanistically, the exact coagulant activity of MSC-derived EVs and the factors regulating this process remain poorly understood. Consequently, because high-quality clinical evidence is still lacking, it remains unclear whether prophylactic administration of anticoagulant drugs is necessary or effective for patients prior to receiving systemic therapeutic EV infusions.

The procoagulant potential of a therapeutic EV batch might be dictated by upstream manufacturing parameters and downstream processing. Common isolation methodologies - such as differential ultracentrifugation, size-exclusion chromatography (SEC), or tangential flow filtration (TFF) - alter the retention of proteins and co-isolate distinct vesicle populations. For instance, compared to EVs harvested at lower centrifugal forces (20,000×g), vesicle fractions isolated via high-speed ultracentrifugation (100,000×g) exhibit significantly lower overall coagulant activity (Nielsen et al., 2018). Furthermore, while extensive washing of the pellet blocks bulk protein content and reduces absolute TF concentration, it does not fundamentally alter the intrinsic procoagulant behavior of the remaining vesicle core (Nielsen et al., 2018).

This processing-dependent risk is compounded by structural heterogeneity and inherent cargo variations of distinct EV subtypes. Vesicle biogenesis pathways create highly diverse populations, spanning apoptotic bodies, microvesicles, and exosome-enriched fractions. Large microvesicles and apoptotic bodies typically carry higher surface concentrations of PS and transmembrane full-length TF isoforms, rendering them highly procoagulant (Muhsin-Sharafaldine et al., 2016). Conversely, purified, exosome-enriched fractions may offer a distinct safety advantage due to a lower baseline thrombogenic signature. However, lessons from cellular counterparts and the transition of EVs from physiological to pathological conditions indicate that both TF encryption-decryption conformation changes and vesicle membrane compositions are highly dynamic and context-dependent. For example, while healthy, full-term UC-MSC-derived EVs lack baseline surface TF, they still support functional procoagulant activity in platelet-free plasma due to a persistent ∼3% baseline exposure of surface PS (Silachev et al., 2019).

Mechanistically, the absolute thrombogenic risk of a therapeutic EV drug is a multi-parametric phenomenon. While TF^+^ EVs represent a small fraction of circulating vesicles under physiological conditions, anionic phospholipids like PS activate TF through a conformational process known as decryption. Exposed surface PS and active TF act synergistically to accelerate the extrinsic coagulation cascade, initiating downstream thrombin generation.

To safely advance cell-free therapies into clinical trials, robust safety assays evaluating hemocompatibility are crucial. Notably, our *in vivo* comprehensive toxicity assays evaluating the safety of human AT- and UC-MSC-derived EVs in mice, rats, and rabbits demonstrated a significantly lower thrombogenic risk profile compared to MSC counterparts when intravenously administered in the absence of anticoagulant prophylaxis (Nguyen et al., 2025; Dinh et al., 2025). However, due to the current lack of robust clinical evidence and the highly heterogeneous nature of diverse EV therapeutics, integrated multi-parametric approaches must be established as standard practice in alignment with evolving international characterization frameworks, such as the Minimal Information for Studies of Extracellular Vesicles (MISEV) guidelines. These quality control pipelines should integrate the quantitative profiling of procoagulant factors on therapeutic EV products with functional tracking methodologies (e.g., FXa generation, chromogenic thrombin monitoring, or viscoelastometric ROTEM/TEG profiling). Importantly, rigorous post-infusion monitoring of D-dimer kinetics and clinical thromboembolic adverse events is essential to ensure patient safety and to provide high-quality data for future clinical research.

Overall, while these data suggest that purified exosomes may offer distinct clinical advantages over larger vesicles owing to their lower baseline coagulant activity, and that naïve EVs are inherently less prothrombogenic than their cellular counterparts, further rigorous investigation remains essential to fully elucidate how tissue origin, upstream culture configurations, and downstream purification unit operations shape their final hemocompatibility profiles.

## 6 Preventing and monitoring thrombosis for cell- and EV-based therapy

As TF-expressing cells and potentially EVs induce IBMIR and VTE in patients after infusion, prevention and treatment strategies should be implemented to prevent adverse events and improve therapeutic efficacy. These include postinfusion monitoring of coagulation parameters, prophylaxis and treatment of thrombosis (Figure 4D).

Table 4 presents the transplantation protocols and postinfusion monitoring of coagulation parameters for patients receiving cell therapy. Increased D-dimer in serum is often observed in the first few days (Moll et al., 2015; Iglesias et al., 2021; Perlee et al., 2018). Coagulation parameters, including the prothrombin time (PT), partial thromboplastin time (PTT), and thrombin time (TT), are also frequently analyzed, but they remained stable in most studies (Hoang et al., 2024; Moll et al., 2015; Aghayan et al., 2022). The platelet number, TATc, and fibrinogen are other useful indicators of coagulopathy that can be assessed (Hoang et al., 2024; Perlee et al., 2018; Baak et al., 2022).

**TABLE 4:** Transplantation protocols and monitoring of thrombosis events.

No.	Publications	Indication	Cell type	Cell dose	Route	Anticoagulant in cell product	Prophylaxis therapy	Laboratory tests after infusion	Clinical manifestations
1	Liang et al. (2010)	Patients with systemic lupus erythematosus, with diffuse alveolar infiltrates in both lung fields	UC-MSCs	1 × 10^6^ cells/kg (n = 1)	IV	Not reported	Methylprednisolone 40 mg per day	Not reported	No thrombosis-related AEs/SAEs
2	Stephenne et al. (2012)	A patient with severe ornithine transcarbamylase deficiency	Human adult liver-derived mesenchymal progenitor cells	30 × 10^6^ cells/kg, two infusions within 14 days (n = 1)	IV	Heparin 10 UI/mL	Tacrolimus 2 mg per day in two divided doses (targeted blood levels of 6–7 ng/mL)	D-Dimer level (markedly increased post infusion)	A partial thrombosis of the intrahepatic portal vein branch occurred during the 2nd infusion; resolution with heparin and coumarinic anticoagulant (5 mg/day)
3	Stephenne et al. (2012)	Patients with intermediate type I/II Crigler-Najjar syndrome (n = 1) and glycogenosis Type 1a (n = 1)	Human adult liver-derived mesenchymal progenitor cells	In total 2.2 × 10^9^ and 3 × 10^9^ cells, seven infusions over 2 and 3 days, respectively (n = 1 each)	IV	Heparin 10 UI/mL	- Bivalirudin 1.75 mg/kg during cell infusion and 0.25 mg/kg between cell infusions - Solumedrol 80 mg before cell infusion - Tacrolimus (targeted blood levels of 6–8 ng/mL)	- D-Dimer level (increased post infusion) - Coagulation tests (increased partial thromboplastin time and thrombin time)	No thrombotic or hemorrhagic events, normal portal flow using liver Doppler ultrasound
4	Moll et al. (2012)	Patients with acute GvHD refractory to standard therapy (n = 30) and tissue injury after HSCT (n = 14)	BM-MSCs	1.0–3.0 × 10^6^ cells/kg, up to five infusions (n = 44)	IV	Cells in heparinized syringe	- Myeloablative conditioning (n = 28) or reduced-intensity conditioning (n = 16) - GvHD prophylaxis (n = 44)	- D-Dimer level (not increased post infusion) - Complement activation maker C3a and thrombin-antithrombin complex (TAT) level (increased post infusion)	No reported AEs/SAEs
5	Lublin et al. (2014)	Patients with multiple sclerosis	Placenta-derived cells (PDA-001)	−150 × 10^6^ cells/infusion; one infusion (n = 6) −600 × 10^6^ cells/infusion; four infusions (n = 6)	IV	Not reported	Not reported	Not reported	A patient had grade 2 superficial thrombophlebitis; resolved without requiring a change in study medication
6	Moll et al. (2015)	Patients with graft-versus-host disease (GvHD; n = 7) and hemorrhagic cystitis (HC; n = 1)	DSCs (placenta-derived decidual stromal cells)	2.0 × 10^6^ cells/mL, two infusions (n = 8)	IV	Cells in heparinized syringe	Myeloablative conditioning (n = 2) or reduced-intensity conditioning (n = 6) and GvHD prophylaxis	- D-Dimer level (increased between 3 and 24 h post infusion) - Coagulation tests (unchanged)	No major adverse thrombotic events
7	Gao et al. (2015)	Patients with ST-elevation acute myocardial infarction	WJ-MSCs	6 × 10^6^ cells (n = 116)	Local delivery	Heparin 10 U/mL	Not reported	Not reported	No thrombosis-related AEs/SAEs
8	Liang et al. (2015)	Laryngeal carcinoma patient with radiation myelitis	UC-MSCs	5.2 × 10^7^ cells iv and 1.1 × 10^7^ cells intrathecal (n = 1)	IV and intrathecal	Heparin	Not reported	Not reported	No reported AEs/SAEs
9	Wu et al. (2017)	Patients with renal chronic kidney disease	UC-MSC	1.0 × 10^6^ cells/kg (n = 2)	IV	Heparin 10 U/mL	- LMWH 2500 IU, subcutaneous - methylprednisolone sodium succinate 40 mg, iv - Promethazine hydrochloride 25 mg, im	- D-Dimer level (increased post infusion) - Coagulation tests (unchanged)	Venous clots at the proximal end of the puncture site; resolution with urokinase (2 × 10^5^ U, twice per day, iv) and warfarin (2.5 g, once per day, oral)
10	Liang et al. (2017)	Patients with liver cirrhosis caused by autoimmune diseases	BM-, UC-, CB-MSCs	1 × 10^6^ cells/kg (n = 26)	IV	Heparin	Low-dose steroids and/or immunosuppressive medications and/or ursodeoxycholic acid and/or other drugs to relieve the symptoms of cirrhosis	Not reported	No thrombosis-related AEs/SAEs
11	Gupta et al. (2017)	Patients with critical limb ischemia due to Buerger’s disease	BM-MSCs (Stempeucel®)	−1 × 10^6^ cells/0.5 mL/kg (n = 36) −2 × 10^6^ cells/0.5 mL/kg (n = 36)	Intramuscular	Not reported	Not reported	Not reported	No thrombosis-related AEs/SAEs
12	Perlee et al. (2018)	Healthy men	AD-MSC	0.25, 1, and 4 × 10^6^ cells/kg (n = 8 each)	IV	Not reported	Lipopolysaccharide 2 ng/kg	- D-Dimer level (increased post infusion) - TAT level (increased post infusion)	​
13	Liang et al. (2018)	Patients with autoimmune disease	BM- and UC-MSCs	1 × 10^6^ cells/kg (n = 404)	IV	Heparin	Not reported	Not reported	No thrombosis-related AEs/SAEs
14	Ringden et al. (2018)	Patients with severe acute graft-versus-host disease	Placenta-derived cells (PDA-001)	−2 × 10^6^ cells/kg (n = 17) −1 × 10^6^ cells/kg, two infusions, 1 week apart (n = 21)	Central venous line	Cells in heparinized syringe	The central venous line was flushed with 12.5–50, I.E., heparin/mL before and after cell infusion	Not reported	No thrombosis-related AEs/SAEs
15	Coppin et al. (2020)	Patients with Crigler-Najjar syndrome (I or II) or urea cycle disorder	HepaStem	12.5 ×10^6^ cells/kg (n = 4), 50 ×10^6^ cells/kg (n = 2), and 200 ×10^6^ cells/kg (n = 5)	IV	Heparin 10 I.U./5 × 106 cell/mL	- Bivalirudin 1.75 mg/kg/h during cell infusion and 0.25 mg/kg between cell infusions - Solumedrol 2 mg/kg before cell infusion - Basiliximab on day 0 days 4 (5–20 mg/day depending on body weight)	D-Dimer level (increased post infusion until day 7)	Portal vein pressure: mild increase
16	Sánchez-Guijo et al. (2020)	Patients with severe SARS-CoV-2 pneumonia requiring mechanical ventilation	AT-MSC	1.0 × 10^6^ cells/kg, three infusions, 2–3 days apart (n = 13)	IV	Not reported	LMWH and corticosteroids before and after the infusion	D-Dimer: decreased after 5 days of the first infusion	Not reported
17	Saleh et al. (2021)	Patients with COVID-19	WJ-MSC	In total 150 × 10^6^ cells/patient, three infusions on days 0, 3 and 6 (n = 5)	IV	Heparin	Hydrocortisone 100 mg	D-Dimer (decreased in 4/5 patients after 3, 6 and 14 days of MSC infusion)	Not reported
18	Häberle et al. (2021)	Patients with Severe COVID-19 ARDS	BM-MSCs	10^6^ cells/kg (n = 5); 2–3 infusions	​	Heparin 2000 U/L used in cell culture	Not reported	D-Dimer level and coagulation tests (comparable between the treated and the placebo group)	No thrombosis-related AEs/SAEs
19	Lanzoni et al. (2021)	Patients with COVID-19	UC-MSCs	In total 100 ± 20 × 10^6^ cells/patient, two infusions on days 0 and 3 (n = 12)	IV	Heparin	LMWH (Enoxaparin 1 mg/kg twice daily)	Not reported	No thrombosis-related AEs/SAEs
20	Iglesias et al. (2021)	Patients with Severe COVID-19 ARDS	UC- MSCs (CBCells BioTechnology)	10^6^ cells/kg (n = 5)	IV	Not reported	Not reported	- D-Dimer level: increased after 24 h post infusion and then decreased	No thrombosis-related AEs/SAEs
21	Sadeghi et al. (2021)	Patients with COVID-19 ARDS	Placenta-derived decidua stromal cells (DSCs)	10^6^ cells/kg, one or two infusion(s) (n = 10)	IV	Cells in heparinized syringe	−10 mg chlorphenamine (10 mg/mL) or 100 mg hydrocortisone before infusion - LMWH (1000 IU/mL) before and after infusion	Not reported	Not reported
22	Attar et al. (2022)	Patients with acute myocardial infarction	WJ-MSCs	1 × 10^7^ cells (n = 130)	Intracoronary	Heparin	Not reported	Not reported	Not reported
23	Baak et al. (2022)	Patients with perinatal arterial ischemic stroke	BM-MSCs	45–50 × 10^6^ cells (n = 10)	Intranasal	Not reported	Not reported	Thrombocyte (increased after 1 day post MSC administration)	No thrombosis-related AEs/SAEs
24	Monsel et al. (2022)	Patients with COVID-19-associated ARDS	UC-MSCs	10^6^ cells/kg, three infusions on days 1, 3, 5 (n = 45)	IV	2 IU/mL heparin used in cell culture	Not reported	D-Dimer level (comparable between the treated and the placebo group)	No thrombosis-related AEs/SAEs
25	Araldi et al. (2022)	Patient with moderate to critically ill COVID-19	Human immature dental pulp stromal cells (NestaCell®)	In total 2 × 10^7^ cells/patient, four infusions on days 1, 3, 5 and 7 (n = 45)	IV	Not reported	LMWH (Enoxaparin)	D-Dimer level and coagulation tests (comparable between the treated and the placebo group)	No thrombosis-related AEs/SAEs
26	Aghayan et al. (2022)	Patients with COVID-19 ARDS	Placenta-derived mesenchymal stem cells (PL-MSCs)	10^6^ cells/kg (n = 10)	IV	2 IU/mL heparin used in cell culture	Not reported	Coagulation tests prothrombin time (PT) and partial thromboplastin time (PTT) (unchanged)	No thrombosis-related AEs/SAEs
27	Sharma et al. (2022)	Patients with COVID-19	Placenta- and umbilical cord blood-derived MSCs	10^7^ cells/kg, two infusions on days 1 and 4 (n = 10)	IV	Not reported	- LMWH - Methylprednisolone	D-Dimer: increased from Day 1 to Day 6 and then sharply decreased	No thrombosis-related AEs/SAEs
28	Hoang et al. (2024)	Patients with stroke (n = 32) Patients with frailty (n = 19)	UC-MSCs	1.5 × 10^6^ cells/kg, two infusions on days 1 and 90 (n = 51)	IV (n = 35) Intrathecal (n = 16)	Not reported	−4,000 Ui Lovenox or 10 mg Xarelto −40 mg Solumedrol	D-Dimer level and coagulation tests (transiently increased D-Dimer in the IV infused group)	No thrombosis-related AEs/SAEs

Abbreviations: adipose tissue (AT), adverse event (AE), bone marrow (BM), cord blood (CB), dental stem cell (DSC), extracellular vesicles (EVs), graft-versus-host disease (GVHD), low-molecular-weight heparins (LMWH), intravenous (IV), mesenchymal stem/stromal cells (MSCs), serious adverse event (SAE), thrombin–antithrombin (TAT), umbilical cord (UC), Wharton jelly (WJ).

Heparin agents, including unfractionated heparin (UFH) and low-molecular-weight heparin (LMWH), represent the most widely documented approaches to mitigating infusion-related thrombotic events (Table 4). Heparin contains heavily negatively charged sulfated glycosaminoglycans that have high affinity for antithrombin III, FXa, and many other proteins (Mulloy et al., 2016). When heparin binds to antithrombin, the complex blocks thrombin and FXa in the coagulant cascade, inhibiting fibrinogen proteolysis into fibrin. As a result, the drug interferes with the formation of new blood clots. Furthermore, heparin is known for its immunosuppressive and antiviral effects (Laner-Plamberger et al., 2021). Heparin can be utilized for cell culture and as an anticoagulant and anti-inflammatory drug for cell-based therapy. In particular, smooth muscle cells were exposed to heparin and its related sulfated polysaccharides for one hour and stimulated with growth factors (PDGF-BB and EGF), resulting in reduced TF production (Xuereb et al., 1999). Lupu et al. reported that heparins significantly induced endothelial cells to secrete the TF pathway inhibitor, an important downregulation factor of the TF/FVIIa complex (Lupu et al., 1999). Heparin was added to the cell culture media of MSCs at a concentration of 2 IU/mL to prevent fibrin clotting in the presence of human platelet lysate (Bieback et al., 2019). Many protocols employ cell products prepared in a heparinized syringe or in a formulation buffer containing heparin and treat patients with heparin to prevent thrombosis events (Moll et al., 2015; Monsel et al., 2022; Attar et al., 2022). Upon injection, heparin can interact with thrombin, antithrombin III, and FXa, thus acting quickly against thrombosis. For prophylactic treatment of thrombosis, patients might receive LMWH, either alone or in combination with immunosuppressive medications (Aghayan et al., 2022; Sadeghi et al., 2021; Araldi et al., 2022). Compared with UFHs, LMWHs contain smaller fragments of glycosaminoglycans and are capable of inhibiting FXa but are less effective against thrombin. Although LMWHs act more slowly than UFHs do, they have a longer half-life, more predictable bioactivity, and potentially fewer side effects (Hirsh, 1998). Some protocols employ bivalirudin and rivaroxaban, which are inhibitors of thrombin and factor Xa, respectively (Coppin et al., 2020; Stephenne et al., 2012). Stephenne et al. reported that a combination of heparin and bivalirudin is more effective at controlling thrombosis than a single drug alone (Stephenne et al., 2012). This protocol could be used for cell types with very high procoagulant activity, such as adult liver-derived MSCs and hepatic stem cells (Coppin et al., 2020; Stephenne et al., 2012). Other potential options for anticoagulants, including other oral factor Xa inhibitors, direct thrombin inhibitors, warfarin, TF inhibitors, thrombolytic therapy, and antiplatelet therapy, should be considered in future research.

It is worth noting that although heparin is commonly used, there is no universal prophylaxis strategy for regenerative medicine products. The clinical deployment of anticoagulation prophylaxis for any cell and EV-containing therapeutics demands systematic risk-benefit assessment and consideration of individual heterogeneity. Indeed, current clinical and preclinical evidence remains insufficient to recommend a single standardized anticoagulant type, dosage, initial administration timing, or treatment duration that applies broadly across all cell- and EV-based therapeutics. Instead, clinical decision-making for each product must establish a delicate balance between product-specific risk parameters, route of administration, and disease background and/or concordance diseases. It is imperative to evaluate product-related variables, including the intrinsic procoagulant activity of the specific tissue source (e.g., adipose or umbilical cord-versus bone marrow-derived cell products or immune cells), upstream cell manufacturing and downstream processing parameters, and biological variations of donors and batches. For route-dependent factors, the intended route of delivery - given that systemic intravascular infusions present distinct hemocompatibility risks compared to localized or encapsulated deliveries.

Depending on the patient’s clinical background, underlying conditions characterized by hyperinflammation and an elevated baseline risk of thromboembolism - such as severe COVID-19, cardiovascular diseases, and systemic autoimmune disorders - necessitate significantly more stringent hemocompatibility testing of cell- or EV-based products and the careful integration of thromboprophylaxis regimens. Consequently, recipient-specific factors must be rigorously weighed. Complex comorbidities, including active malignancies, profound inflammatory states, advanced renal impairment, or pre-existing bleeding risk, mandate heightened clinical vigilance and may warrant strict exclusion criteria to ensure patient safety.

On the other hand, when using thromboprophylaxis drugs, clinicians must account for potential drug-related complications, such as heparin-induced thrombocytopenia (HIT) or bleeding liabilities.

Consequently, rather than adopting a rigid, prescriptive approach, future guidelines should focus on establishing standardized assays to quantify the coagulant activity of blood-contacting living drugs and cell-free therapeutics. Furthermore, developing robust hemocompatibility risk-assessment protocols and consensus recommendations tailored to patient-specific clinical conditions is essential to guide the safe administration of cell- and EV-based products, thereby ensuring optimal safety and efficacy.

## 7 Conclusion and future directions

TF isoforms are multifunctional proteins with pivotal roles in coagulation and cellular regulation. While it is expressed in various cell types that are not in direct contact with blood under physiological conditions, it can be upregulated upon stimulation. Hence, TF and its induced thrombotic activity are critical considerations in regenerative medicine. Several therapeutic approaches, including cell therapy using differentiated ES and iPSC derivatives, MSCs, pancreatic islet cells, and liver cells, as well as organ transplantation, might trigger IBMIR and VTE in patients. Additionally, TF^+^ EVs are present in the body and can be upregulated in response to external stimuli. To enhance the safety of biotherapeutic drugs, addressing TF expression and coagulant activity is crucial. Importantly, medical centers should develop effective IBMIR and VTE prophylaxis and treatment strategies. Most centers rely on their own administration protocols for therapeutic cells, tissues, and EVs. However, high-quality evidence is still lacking for the choice of anticoagulant drugs, their optimal usage, and their effects on therapeutic efficacy. Furthermore, monitoring coagulation parameters is essential, as deep vein thrombosis and pulmonary embolism can be life-threatening. To advance our understanding, comprehensive preclinical studies, well-designed randomized clinical studies, systematic reviews and meta-analyses are needed to evaluate the impact of anticoagulants on their safety and effectiveness. Recommendations for anticoagulant type, dosage, initial administration time and treatment duration are also needed for both prophylactic and therapeutic purposes. Additionally, future clinical research should rigorously monitor postintervention coagulation and detect thrombotic events early.

## Glossary

HNE: 4-hydroxy-2-nonenal
SARS-CoV-2: acute respiratory syndrome coronavirus 2
ADP: adenosine diphosphate
asTF: alternative splicing TF isoform
AT: adipose tissue
ATP: adenosine triphosphate
bFGF: basic fibroblast growth factor
BM: bone marrow
Ca: calcium
cAMP: cyclic adenosine monophosphate
DP: dental pulp
DAG: diacylglycerol
EGF: epidermal growth factor
EPCR: endothelial protein C receptor
ESCs: embryonic stem cells
EVs: extracellular vesicles
FVIIa: factor VIIa
FXa: factor Xa
HUVECs: human umbilical vein endothelial cells
IBMIR: instant blood-mediated inflammatory reactions
iPSCs: induced pluripotent stem cells
IV: intravenous
LMWH: low-molecular-weight heparins
MAPK: mitogen-activating phosphate kinase
MSCs: mesenchymal stem/stromal cells
PD: placental decidua
PDGF: platelet-derived growth factor
PI3: phosphatidyl inositol triphosphates
PM: placenta membrane
PS: phosphatidylserine
PT: prothrombin time
PTT: partial thromboplastin time
ROS: reactive oxidative species
ROTEM: rotational thromboelastometry
TATc: thrombin–antithrombin complex
TEG: thromboelastography
TrxR: thioredoxin reductase
Trx: thioredoxin
TF: tissue factor
TT: thrombin time
UC: umbilical cord
UFH: unfractionated heparins
VEGF: vascular endothelial cell growth factor
VTE: venous thromboembolism
vWF: Willebrand factor
